# Abnormal resting-state cerebral-limbic functional connectivity in bipolar depression and unipolar depression

**DOI:** 10.1186/s12868-019-0508-6

**Published:** 2019-06-17

**Authors:** Chang Liu, Weidan Pu, Guowei Wu, Jie Zhao, Zhimin Xue

**Affiliations:** 1 Department of Psychiatry, Brains Hospital of Hunan Province, Changsha, Hunan People’s Republic of China; 2 Post-Doctoral Research Mobile Station, Hunan University of Traditional Chinese Medicine, Changsha, Hunan People’s Republic of China; 3 Mental Health Institute, Second Xiangya Hospital, Central South University, Changsha, Hunan People’s Republic of China; 4 State Key Laboratory of Medical Genetics, Central South University, Changsha, Hunan People’s Republic of China; 5 Medical Psychological Center, Second Xiangya Hospital, Central South University, Changsha, Hunan People’s Republic of China; 6 The China National Clinical Research Center for Mental Health Disorders, National Technology Institute of Psychiatry, Key Laboratory of Psychiatry and Mental Health of Hunan Province, 139 Middle Renmin Road, Changsha, 410011 Hunan People’s Republic of China

**Keywords:** Depression, Cerebral-limbic, Functional activity, Resting-state

## Abstract

**Background:**

Distinctive patterns of functional connectivity (FC) abnormalities in neural circuitry has been reported in patients with bipolar depression (BD) and unipolar depression (UD). However, it is unclear that whether this distinct functional connectivity patterns are diagnosis specific between BD and UD. This study aimed to compare patterns of functional connectivity among BD, UD and healthy controls (HC) and determine the distinct functional connectivity patterns which can differentiate BD from UD.

**Method:**

Totally 23 BD, 22 UD, and 24 HC were recruited to undergo resting-state fMRI scanning. FC between each pair of brain regions was calculated and compared among the three groups, the associations of FC with depressive symptom were also analyzed.

**Results:**

Both patient groups showed significantly decreased cerebral-limbic FC located between the default mode network [posterior cingulated gyrus (PCG) and precuneus] and limbic regions (hippocampus, amygdala and thalamus) than HC. Moreover, the BD group exhibited more decreased FC mainly in the cortical regions (middle temporal gyrus, PCG, medial superior frontal gyrus, inferior occipital gyrus and superior temporal gyrus), but the UD group is more associated with limbic alterations. These decreased FCs were negatively correlated with HAMD scores in both BD and UD patients.

**Conclusions:**

BD and UD patients demonstrate different patterns of abnormal cerebral-limbic FC, reflected by decreased FC within cerebral cortex and limbic regions in BD and UD, respectively. The distinct FC abnormal pattern of the cerebral-limbic circuit might be applied as biomarkers to differentiate these two depressive patient groups.

## Background

Major depressive disorder (unipolar disorder, UD) and bipolar disorder (BD) are both severe, episodic, life-long mood disorders. Unlike unipolar disorder, bipolar disorder is characterized by emotional instability interspersed with depressive episode and (hypo-) manic episode [1–3]. Moreover, if the patients with bipolar disorders present with a major depressive episode as their first mood episode, it is extremely difficult to discriminate the bipolar depression from the unipolar depression [4]. Within the first year to seek treatment, close to 60% of BD patients are misdiagnosed with UD [5, 6], and antidepressant treatment for these patients lead to poor clinical and functional outcome, and enhance the burden of the family and society [7–9]. However, the pathogenic mechanisms of unipolar and bipolar depression are largely unknown, so diagnostic boundaries are difficult to define. Thus, a study aiming to establish objective measures to distinguish BD from UD can help improve the current diagnostic methods, eventually leading to better treatment outcomes.

Convergent studies from neuroanatomy, neurochemistry and functional magnetic resonance imaging (fMRI) have widely demonstrated neurobiological correlates of mood disorder [10–13]. Functional neuroimaging studies directly comparing bipolar and unipolar depression suggest different patterns of neuronal defect between these two neuropsychiatric disorders [14]. For instance, a few studies comparing pattern of brain activation during emotion processing and emotion regulation task in BD and UD patients reported over-activation of the amygdala in BD patients [15], elevated dorsal anterior cingulate cortical activity [16] and reduced activity of ventral lateral prefrontal cortex (VLPFC) and amygdala in UD patients [17]. In addition, reduced effective connectivity between bilateral amygdala and ventral medial prefrontal cortex (VMPFC) has been only seen in UD patients [18]. Resting-state fMRI studies have shown increased activity in thalamus, sub-cingulate cortex [19] in UD, and altered activity in fronto-limbic regions in BD [20]. In parallel, most consistent morphological finding is the abnormally enlarged volume of putamen and caudate in bipolar depression relative to unipolar depression [13, 21]. Moreover, glutamate-related magnetic resonance spectroscopy (MRS) measures have reported that Glx (composed mainly of glutamate and glutamine) reduced in prefrontal and subcortical regions in unipolar depression, but increased in all mood states in bipolar disorder [21, 22]. Taken together, these evidence suggest that the neuropathology of depression involves dysfunctions within a neuroanatomical circuit including the frontal cortex and limbic structures [23, 24], but the specific pattern of neural changes are different between unipolar and bipolar depression in this circuit. These observed neuronal deficits in BD differed from UD is of potential meanings for distinguishing these two diseases. However, most existing studies had a major shortcoming that the recruited BD patients included a manic phase, depressive phase or remission phase, which cannot rule out the interference of various disease states [15, 19].

Therefore, in this current study, we recruited the BD and UD patients who were all at the depressive episode (met the criteria for a current episode for major depression with Hamilton Depression Rating Scale [HAMD ≥ 17] and Young Mania Rating Scale [YMRS < 6]) and compared patterns of functional connectivity among BD, UD and healthy controls (HC). This study aimed to explore if the distinct functional connectivity patterns were diagnosis specific between BD and UD.

## Methods

### Study participants and recruitment

Twenty-three bipolar depression and Twenty-two unipolar depression patients were recruited from the inpatient and outpatient units of the Department of Psychiatry at the Second Xiangya Hospital, Central South University, Changsha, China. All patients were diagnosed with bipolar depression disorder or major depression disorder according to the Structured Clinical Interview-Patient version for DSM-IV. Inclusion criteria included: age between 18 and 45 years old; Han Chinese ethnicity; finished ninth grade or higher levels of education; and sufficient understanding and expressive capacity; total score ≥ 17 on the 17-item Hamilton Depression Rating Scale (HAMD) and total score < 6 on the Young Mania Rating Scale (YMRS). Exclusion criteria included: severe learning disability; a current diagnosis of substance-induced psychosis; alcohol use within 24 h prior to interview and scanning; a history of brain trauma or neurological disease; left-handedness; previous electroconvulsive therapy and any other contraindications to MRI. All subjects were taking antipsychotics at the time of the study. Benzodiazepine treatment, if any, was stopped for 24 h prior to scanning. Twenty-four healthy controls were recruited from the Changsha city area. The inclusion and exclusion criteria were the same as those for depression disorder patients except that controls should not meet the DSM-IV criteria of Axis-I psychiatric disorder. All HC were well matched with the two patient groups in terms of gender (*χ*^2^= 0.185, *p* = 1.679) and years of education (*t* = 0.911, *p* = 0.126), except for age (*t* = − 2.064, *p* = 0.014). The two patient groups were well matched in HAMD scores. All subjects signed informed consent to participate in the study. The study was approved by the ethics committee of the Second Xiangya Hospital of Central South University.

### Assessments and procedures

All subjects were assessed for cognitive functions with Information and Digit symbol coding subsets of Wechsler Adult Intelligence Scale (WAIS). Subjects’ demographic including age, sex, years of education were recorded. Clinical information including diagnosis and duration of illness in patients were recorded. All patients were assessed using the Hamilton Depression Rating Scale (HAMD) [25] and Young Mania Rating Scale (YMRS) [26]. All patients were received the MRI scan within 1 week after diagnosis. Moreover, before the scan, all patients were assessed by the 17-item Hamilton Depression Rating Scale (HAMD) and the Young Mania Rating Scale (YMRS) to make sure the patients were in the depressive episode at the scanning time.

### fMRI date acquisition

Imaging data were collected using a 3.0-Tesla Philips Achieva whole-body MRI scanner (Philips, The Netherlands) in a session of 8 min 26 s duration, in which 250 volumes were acquired. Images were obtained using a gradient echo echo-planar imaging (EPI) sequence with the following parameters: repetition time (TR) = 2000 ms, echo time (TE) = 30 ms, flip angle = 90°, matrix 64 × 64, slice thickness = 4 mm, gap = 0 mm, slices = 36.

### Image processing

Before functional images preprocessing, the first 10 volumes were removed to allow for scanner stabilization and the subjects’ adaptation to the environment. Each subject’s functional image was preprocessed using the SPM8 (University College London, UK; http://www.fil.ion.ucl.ac.uk/spm) and DPARSF (Data Processing Assistant for resting-state fMRI). The remaining functional images were first corrected for within–scan acquisition time differences between slices, and realigned to the middle volume to correct for inter-scan head motions. Subsequently, the functional images were resampled to the Montreal Neurological Institute echo-planar imaging template (each voxel was resampled to 3*3*3 mm^3^) during DARTEL normalization. After normalization, the Blood Oxygenation Level Department (BOLD) signal of each voxel was first detrended and then passed through a band-pass filter (0.01–0.08 Hz) to reduce low-frequency drift and high-frequency physiological noise. Finally, nuisance covariates including head motion parameters, global mean signals, white matter signals and cerebrospinal fluid signals were regressed out from the Blood Oxygenation Level Dependent signals.

Since recent studies demonstrated that head motion may have both noisy and neuronal effect on functional connectivity measures [27, 28], we performed ‘scrubbing’ to ensure that head-motion artifacts are not driving the observed effects. An estimate of head motion at each time-point was calculated as the frame-wise displacement (FD). Following previous studies as reported by Power et al. [29], any image with FD > 0.5 was removed and replaced by a linear interpolation. The mean absolute FD between BD, UD and HC did not differ significantly [mean BD: 0.18 (SD = 0.06), mean UD: 0.18 (SD = 0.06), mean HC: 0.16 (SD = 0.07), N.S.].

### Data analysis

#### Demographic, clinical and behavioral data

Demographic variables across the three groups were compared with the one-way analysis of variance (ANOVA) or independent sample *t* tests for continuous variables and Chi-square tests for categorical variables. Analysis of covariance adjusted for age differences between groups was used for comparison of cognitive performance across the three groups.

#### Imaging data

Before calculating functional connectivity, the subject-specific components related to movement and physiological noise (e.g. cardiac cycle, respiration) were removed to reduce the overall data variance. Each brain was divided into 90 regions by using the Automated Anatomical Labeling (AAL) template [30]. Given that we were specifically interested on the fronto-limbic circuit, functional connectivity maps between each cerebral region were obtained for each participant using an Automated Anatomical Labeling (AAL) template dividing each brain into 90 regions which only included the cerebral regions (by excluding the cerebellar regions). To increase the statistical reliability of the results, we used a whole-brain scheme, but not the seed-to-seed methodology, to assess the group differences in the functional connectivity among three groups. For all 90 brain regions we calculated the average fMRI time courses of all components after removing components of artifacts. Then the correlations between the signals were computed as the functional connectivity of the brain areas. Between-group differences were tested using two-sample *t* tests with false discovery rate (FDR) correction at the threshold with *p* < 0.05. Pearson analysis was used to evaluate the relationship between functional connectivity with group difference and the HAMD scores (Table 1).

**Table 1:** Names and abbreviations of the regions of interest used in this study

Regions	Abbr.	Regions	Abbr.
Amygdala	AMYG	Hippocampus	HIP
Thalamus	THA	Angular gyrus	ANG
Inferior temporal gyrus	ITG	Medial superior frontal gyrus	SFGmed
Superior temporal gyrus	STG	Inferior occipital gyrus	IOG
Calcarine cortex	CAL	Superior occipital gyrus	SOG
Supramarginal gyrus	SMG	Superior orbitofrontal cortex	ORBsup
Putamen	PUT	Pallidum	PAL
Middle temporal gyrus	MTG	Posterior cingulate gyrus	PCG
Precuneus	PCUN		

## Results

### Demographics, clinical, and behavioral data

Demographic information and clinical characteristics were presented in Table 2. There was no significant difference in gender, years of education among the three groups. The patient groups did not differ significantly in HAMD scores and the medication dosage. However, the UD group had longer illness duration and was older than the BD group. And, the BD patients showed significantly lower score on the WAIS-Digit symbol relative to UD patients.

**Table 2:** Demographic and clinical profiles of bipolar depression patients, unipolar depression patients and healthy controls

Characteristics (mean ± SD)	BD (n = 23)	UD (n = 22)	HC (n = 24)	Analysis	
				F/χ^2^	*p*
Age (years)	24.67 ± 6.17	27.57 ± 4.06	25.82 ± 5.42	− 2.064	0.014^a^
Education (years)	11.41 ± 2.69	11.84 ± 2.69	10.78 ± 2.95	1.679	0.126^a^
Sex (male/female)	12/11	12/10	13/11	0.185	0.911^a^
Duration of illness (months)	9.59 ± 4.53	9.59 ± 4.53	–	− 6.518	< 0.001^a^
Chlorpromazine equivalents (mg)	276.12 ± 245.86	259.00 ± 249.79	–	0.333	0.702^b^
HAMD	21.57 ± 4.67	20.05 ± 5.78	–	0.506	0.671^b^
YMRS	15.84 ± 7.7	14.29 ± 6.53	–	1.084	0.841^b^
WAIS-digit symbol	53.56 ± 12.08	69.50 ± 14.37	79.07 ± 11.44	27.365	< 0.001^b^

*p *< 0.05

*HC* healthy controls, *BD* bipolar depression, *UD* unipolar depression, *HAMD* Hamilton Rating Scale for Depression, *YMRS* Young Mania Rating Scale

^a^Analysis of variance

^b^Two-sample *t* tests

### Functional connectivity

**Fig. 1:**
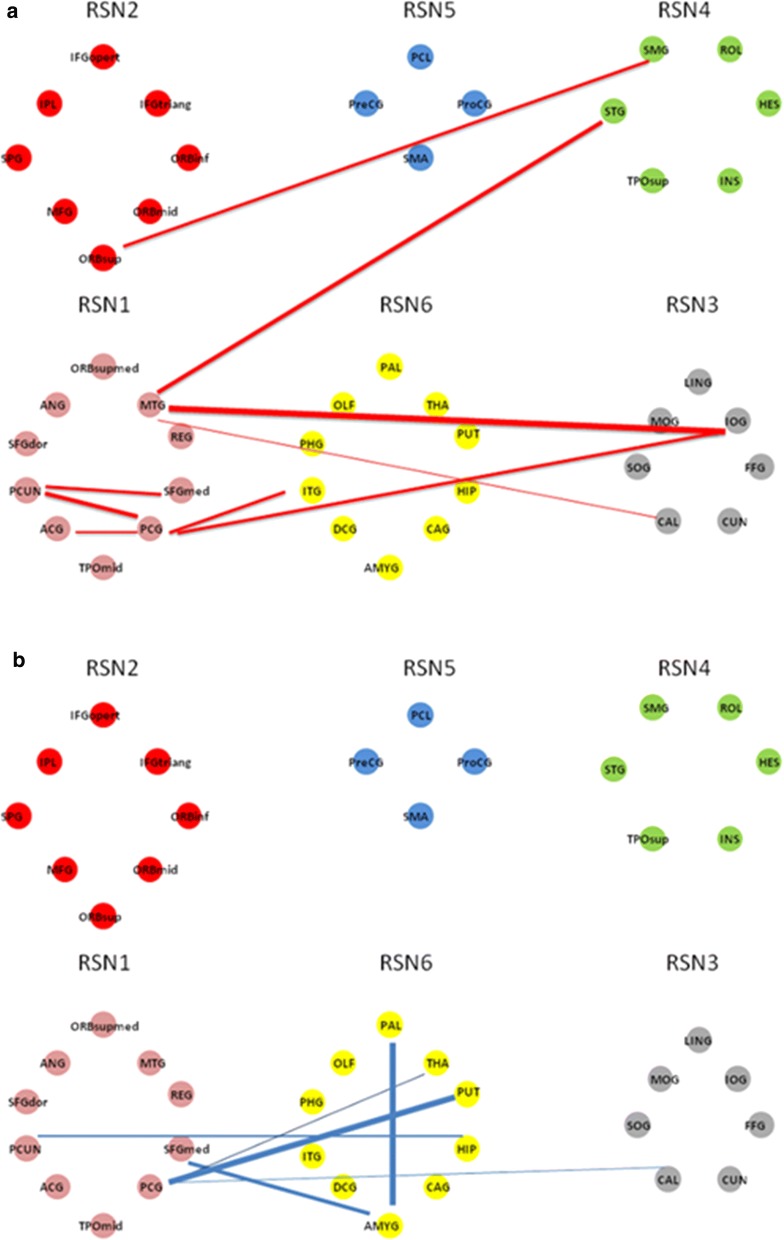
**a **Functional connectivity differences between bipolar depression (BD) and healthy controls (HCs). **b** Functional connectivity differences between unipolar depression (UD) patients and healthy controls (HCs). The red lines indicate links that are decreased in networks of the BD patients compared to HCs groups, while blue lines are links that are decreased in UD compared to HCs groups. The widths of the lines are proportional to the mean strength of functional connections value. Based on our prior work [45], functional connectivity was mapped on six communities corresponding to six resting-state networks (RSN): RSN1-default mode network (DMN), RSN2-attention network, RSN3-visual recognition network, RSN4-auditory network, RSN5-sensory-motor areas, RSN6-subcortical network

After FDR correction (*p* < 0.05), there were no significant differences in the functional connectivity between patients with BD and healthy subjects, as well as between patients with UD and healthy subjects. However, at a uncorrected threshold with *p* < 0.001, compared to the HC group (Table 3 and Fig. 1), the BD and UD groups both exhibited decreased cerebral-limbic FC located between the default mode network (DMN) [posterior cingulated gyrus (PCG) and precuneus] and limbic regions (hippocampus, amygdala and thalamus); Moreover, the BD group exhibited more decreased FC mainly in connected regions within cerebral cortex [middle temporal gyrus (MTG), PCG, medial superior frontal gyrus (SFGmed), inferior occipital gyrus (IOG) and superior temporal gyrus (STG)], while UD group mainly showed decreased FC with the limbic regions [putamen (PUT), pallidum (PAL), hippocampus (HIP), amygdala (AMYG), thalamus (THA)]. Increased FC was found between the left PCG and left superior occipital gyrus only in BD group relative to HC.

**Table 3:** Functional connectivity differences in patients with bipolar depression and unipolar depression relative to healthy subjects

Connections	*t*	*p*	Connections	*t*	*p*
BD < HC					
PCG.R-IOG.L	4.529	0.000273	SFGmed.L-PCUN.R	4.546	0.000283
PCG.R-ITG.R	4.615	0.000306	CAL.R-MTG.L	3.384	0.000162
ACG.R-PCG.R	4.062	0.000220	PCG.R-PCUN.R	5.006	0.000648
MTG.R-IOG.L	5.035	0.000976	ORBsup.R-SMG.L	5.010	0.000707
STG.R-MTG.L	3.026	0.000146			
BD > HC					
PCG.L-SOG.L	4.337	0.000496			
UD < HC					
PCG.R-THA.R	2.922	0.000115	AMGY.R-PAL.L	5.024	0.000951
PCG.L-CAL.R	4.639	0.000388	PCG.R-PUT.R	5.022	0.000919
PCUN.L-HIP.R	5.003	0.000612			
SFGmed.R-AMGY.L	5.015	0.000882			

The abbreviations please see the Table 1

*BD* bipolar depression, *UD* unipolar depression

### Clinical correlations

As shown in the Table 4, the connections of the right inferior temporal gyrus with the right posterior cingulate gyrus (r = − 0.440, *p* = 0.032) were found to be negatively correlated with HAMD scores in BD group. We also found the connections of the right posterior cingulate gyrus with the right thalamus (r = − 0.611, *p* = 0.003) and that of the left pallidum with the right amygdala (r = − 0.649, *p* = 0.004) were negatively with HAMD scores in UD group. All the correlation analyses were not corrected (i.e., *p* < 0.05, uncorrected).

**Table 4:** Correlations of abnormal functional connectivity with clinical measurements in BD and UD

Functional connectivity	Clinical variables	r	*p*
BD			
PCG.R-ITG.R	HAMD scores	− 0.440	0.032
UD			
AMGY.R-PAL.L	HAMD scores	− 0.649	0.004
PCG.R-THA.R	HAMD scores	− 0.611	0.003

The abbreviations please see the Table 1

*HAMD* Hamilton Depression Rating Scale, *BD* bipolar depression, *UD* unipolar depression

## Discussion

The present study compared the patterns of functional connectivity between patients with bipolar disorder depression, unipolar depression and healthy controls. Both patient groups exhibited significantly decreased cerebral-limbic FC located between the DMN (PCG and angular gyrus) and limbic regions (hippocampus, amygdala and thalamus) than controls. Notably, while patients with BD exhibited more decreased FC mainly in connected regions within cerebral cortex (MTG, PCG, SFGmed, IOG and STG), UD mainly showed decreased FC within the limbic regions. Our findings contribute to the understanding of the neurophysiology of these psychiatric illnesses that the differential abnormal patterns of functional connectivity in the cerebral-limbic circuit may be of potentials for distinguishing BD from UD.

Compared to healthy controls, we found decreased FC between the DMN (PCG and angular gyrus) and limbic regions (hippocampus, amygdala and thalamus) in both BD and UD patients. The DMN and limbic regions are commonly regarded as key brain regions in depression, with structural and functional abnormalities [14, 31] being consistently observed in previous studies. Increased depression-related DMN functional connectivity [19, 32, 33] and decreased resting-state connectivity with the DMN regions in UD were reported by several studies [34]. For the BD, decreased task-based activity and increased resting-state activity in DMN were found in previous studies [15, 35]. These two depressive patients have also been found to have structural and functional abnormalities in limbic regions (hippocampus, amygdala and thalamus) [36–38]. Thus, the shared reduced functional connectivity in these regions might explain the overlapping clinical depressive symptoms between BD and UD, as supported by our finding showing the significant associations between the reduced FC and the HAMD scores in both patient groups. Our study extends the prior evidence to that abnormal resting-state functional connectivity between the DMN and limbic regions is implicated in the pathophysiology of depression in both BD and UD.

Despite the common pattern of reduced FC between the DMN and limbic regions in the cerebral-limbic circuit, BD patients mainly exhibited decreased FC in connected regions within cerebral cortex (MFG, PCG, SFGmed, IOG and STG), whereas the UD patients mainly showed decreased FC with the limbic regions (PUT, PAL, HIP, AMYG, THA). Our findings are consistent with a recently proposed BD model highlighting the dysfunctional cerebral cortex [39, 40], suggesting that bipolar depression might be associated with functional abnormalities in neural systems supporting cognitive processing, whereas major depression might be associated with functional abnormalities predominantly within the regions involving emotion regulation.

Clinically, BD patients are often distinguished from UD patients with manic episode and cognitive impairments [41]. In addition, BD patients always exhibit poorer neuropsychological performance than UD [42], consistent with our finding that BD patients showed significantly lower score on the WAIS-Digit symbol relative to UD patients. Therefore, abnormal functional connectivity of cerebral cortex in BD but not UD might explain the clinical phenomenon that cognitive impairment are more severe in BD relative to UD [43]. The limbic region is a central part of the ‘‘emotional brain’’ circuitry responsible for the processing and regulation of emotion [14]. Our findings suggest that the major depressive disorder may be more associated with the abnormalities of the emotional circuitry linking to the limbic structures. Most importantly, the different patterns of abnormalities in the cerebral-limbic circuit may be a potential biomarker for differentiate the BD from UD.

Several limitations of this study need comment. First, age and duration of illness were not well balanced in three groups in this study which may influence the result. To better validate our main findings, we firstly balanced the age and duration of illness among three groups by deleting 8 BD patients and 4 UD patients, and then compared the functional connectivity in the well-matched samples. We found that the results were similar to our prior findings, suggesting the age and illness of duration had subtle influences on our findings. Secondly, all patients in this study were all receiving medication therapy, which may influence on our findings. Future studies need to verify our findings in patients without medication. Thirdly, owing to the relatively small sample, we found no significant difference in the functional connectivity between patients with BD and healthy subjects, as well as between patients with UD and healthy subjects after FDR correction, future studies using larger samples are needed to replicate our findings. Finally, a multivariate pattern analysis of brain images has been suggested as a better choice to discriminate the neuropsychiatric disorders in previous studies [44], future studies using the multivariate pattern analysis on the cerebral-limbic circuit may help providing further insight into whether the distinct functional connectivity patterns observed in this study are diagnosis specific between BD and UD.

## Conclusions

In summary, in the present study we demonstrated that different patterns of abnormal cerebral-limbic FC in bipolar depression and unipolar depression may be a biomarker for distinguishing BD from UD. This finding suggests that these different types of depression are involved in different neurobiology mechanisms based on our fMRI findings, which is valuable for clinical diagnosis and proper treatment choice for these two types of depression.

## Data Availability

All data generated or analyzed during this study are included in this published article. All of the raw data are stored in Department of Psychiatry, Brains Hospital of Hunan Province and Second Xiangya Hospital, Central South University. The datasets used and/or analyzed during the current study are available from the corresponding author on reasonable request.

## Abbreviations

BD: bipolar depression
UD: unipolar depression
PCA: principal components analysis
fMRI: functional magnetic resonance imaging
FC: functional connectivity
HAMD: Hamilton Depression Rating Scale
DMN: default mode network
PCG: posterior cingulated gyrus
MFG: middle frontal gyrus
SFGmed: medial superior frontal gyrus
IOG: inferior occipital gyrus
STG: superior temporal gyrus
WAIS: Wechsler Adult Intelligence Scale
YMRS: Young Mania Rating Scale
EPI: echo echo-planar imaging
BOLD: Blood Oxygenation Level Department
ANOVA: analysis of variance
PUT: putamen
PAL: pallidum
HIP: hippocampus
AMYG: amygdala
THA: thalamus
ORBmid: middle frontal gyrus, orbital
ITG: inferior temporal gyrus
MTG: middle temporal gyrus
MFG: middle frontal gyrus
OFG: orbito-frontal gyrus
PFC: prefrontal cortex
STG: superior temporal gyrus
OLF: olfactory
HES: Heschl gyrus
CAU: caudate nucleus
REC: rectus gyrus
CAL: calcarine cortex
SOG: superior occipital gyrus
SMG: supramarginal gyrus
ORBsup: superior orbitofrontal cortex
PoCG: postcentral gyrus
ANG: angular gyrus

## References

[1] Green MJ, Cahill CM, Malhi GS (2007). The cognitive and neurophysiological basis of emotion dysregulation in bipolar disorder. J Affect Disord.

[2] Mitchell PB, Malhi GS (2002). The expanding pharmacopoeia for bipolar disorder. Annu Rev Med.

[3] Phillips ML, Kupfer DJ (2013). Bipolar disorder diagnosis: challenges and future directions. Lancet.

[4] Woo YS, Shim IH, Wang H-R, Song HR, Jun T-Y, Bahk W-M (2014). A diagnosis of bipolar spectrum disorder predicts diagnostic conversion from unipolar depression to bipolar disorder: a 5-year retrospective study. J Affect Disord.

[5] Cardoso de Almeida JR, Phillips ML (2013). Distinguishing between unipolar depression and bipolar depression: current and future clinical and neuroimaging perspectives. Biol Psychiat.

[6] Hirschfeld RM, Lewis L, Vornik LA (2003). Perceptions and impact of bipolar disorder: how far have we really come? Results of the national depressive and manic-depressive association 2000 survey of individuals with bipolar disorder. J Clin Psychiatry.

[7] Berk M, Berk L, Moss K, Dodd S, Malhi GS (2006). Diagnosing bipolar disorder: how can we do it better?. Med J Aust.

[8] Rush A, Trivedi M, Wisniewski S, Nierenberg A, Stewart J, Warden D, Fava M (2006). A cute and longer-term outcomes in depressed outpatients requiring one or several treatment steps: a STAR* D report. Am J Psychiatry.

[9] Valentí M, Pacchiarotti I, Bonnín CM, Rosa AR, Popovic D, Nivoli AM, Vieta E (2012). Risk factors for antidepressant-related switch to mania. The Journal of Clinical Psychiatry.

[10] Frye MA, Watz J, Banakar S, O’Neill J, Mintz J, Davanzo P, Fischer J, Chirichigno J, Ventura J, Elman S, Tsuang J, Walot I, Thomas M (2007). Increased anterior cingulate/medial prefrontal cortical glutamate and creatine in bipolar depression. Neuropsychopharmacology.

[11] Lui S, Wu Q, Qiu L, Yang X, Kuang W, Chan RCK, Huang X, Kemp GJ, Mechelli A, Gong Q (2011). Resting-state functional connectivity in treatment-resistant depression. Am J Psychiatry.

[12] Rajkowska G, O’Dwyer G, Teleki Z, Stockmeier CA, Miguel-Hidalgo JJ (2007). GABAergic neurons immunoreactive for calcium binding proteins are reduced in the prefrontal cortex in major depression. Neuropsychopharmacology.

[13] Strakowski SM, Adler CM, DelBello MP (2002). Volumetric MRI studies of mood disorders: do they distinguish unipolar and bipolar disorder?. Bipolar Disord.

[14] Phillips ML, Drevets WC, Rauch SL (2003). Neurobiology of emotion perception I: The neural basis of normal emotion perception. Biol Psychiat.

[15] Almeida JR, Versace A, Hassel S, Kupfer DJ, Phillips ML (2010). Elevated amygdala activity to sad facial expressions: a state marker of bipolar but not unipolar depression. Biol Psychiat.

[16] Bertocci MA, Bebko GM, Mullin BC, Langenecker SA, Ladouceur CD, Almeida JRC, Phillips ML (2012). Abnormal anterior cingulate cortical activity during emotional nback task performance distinguishes bipolar from unipolar depressed females. Psychol Med.

[17] Taylor Tavares JV, Clark L, Furey ML, Williams GB, Sahakian BJ, Drevets WC (2008). Neural basis of abnormal response to negative feedback in unmedicated mood disorders. Neuroimage.

[18] Almeida JR, Versace A, Mechelli A, Hassel S, Quevedo K, Kupfer DJ, Phillips M (2009). Abnormal amygdala-prefrontal effective connectivity to happy faces differentiates bipolar from major depression. Biol Psychiatry.

[19] Greicius MD, Flores BH, Menon V (2007). Resting-state functional connectivity in major depression: abnormally increased contributions from subgenual cingulate cortex and thalamus. Biol Psychiat.

[20] MacMaster F, Carrey N, Langevin L, Jaworska N, Crawford S (2014). Disorder-specific volumetric brain difference in adolescent major depressive disorder and bipolar depression. Brain Imaging Behav.

[21] Hasler G, Northoff G (2011). Discovering imaging endophenotypes for major depression. Molecular Psychiatry.

[22] Yuksel C, Öngür D (2010). Magnetic resonance spectroscopy studies of glutamate-related abnormalities in mood disorders. Biol Psychiat.

[23] Bora E, Harrison BJ, Davey CG, Yucel M, Pantelis C (2012). Meta-analysis of volumetric abnormalities in cortico-striatal-pallidal-thalamic circuits in major depressive disorder. Psychol Med.

[24] Price JL, Drevets WC (2010). Neurocircuitry of mood disorders. Neuropsychopharmacology.

[25] Hamilton M (1960). A rating scale for depression. J Neurol Neurosurg Psychiatry.

[26] Young RC, Biggs JT, Ziegler VE, Meyer DA (1978). A rating scale for mania: reliability, validity and sensitivity. Br J Psychiatry.

[27] Zeng L, Wang D, Fox MD, Sabuncu MR, Hu D, Ge M, Liu H (2014). Neurobiological basis of head motion in brain imaging. Proc Natl Acad Sci USA.

[28] Van Dijk KR, Sabuncu MR, Buckner RL (2012). The influence of head motion on intrinsic functional connectivity MRI. NeuroImage.

[29] Power JD, Barnes KA, Snyder AZ, Schlaggar BL, Petersen SE (2012). Spurious but systematic correlations in functional connectivity MRI networks arise from subject motion. NeuroImage.

[30] Tzouriomazoyer N, Landeau B, Papathanassiou D, Crivello F, Etard O, Delcroix N, Joliot M (2002). Automated anatomical labeling of activations in SPM using a macroscopic anatomical parcellation of the MNI MRI single-subject brain. NeuroImage.

[31] Liu CH, Ma X, Wu X, Zhang Y, Zhou FC, Li F, Tie CL, Jie D, Wang YJ, Yang Z, Wang CY (2013). Regional homogeneity of resting-state brain abnormalities in bipolar and unipolar depression. Prog Neuropsychopharmacol Biol Psychiatry.

[32] Zhou Y, Yu C, Zheng H, Liu Y, Song M, Qin W, Jiang T (2010). Increased neural resources recruitment in the intrinsic organization in major depression. J Affect Disord.

[33] Grimm S, Boesiger P, Beck J, Schuepbach D, Bermpohl F, Walter M, Northoff G (2009). Altered negative BOLD responses in the default-mode network during emotion processing in depressed subjects. Neuropsychopharmacology.

[34] Anand A, Li Y, Wang Y, Wu J, Gao S, Bukhari L, Lowe MJ (2005). Activity and connectivity of brain mood regulating circuit in depression: a functional magnetic resonance study. Biol Psychiat.

[35] Marchand WR, Lee JN, Johnson S, Gale P, Thatcher J (2013). Differences in functional connectivity in major depression versus bipolar II depression. J Affect Disord.

[36] Price JL, Drevets WC (2010). Neurocircuitry of mood disorders. Neuropsychopharmacology.

[37] Price JL, Drevets WC (2012). Neural circuits underlying the pathophysiology of mood disorders. Trends Cogn Sci.

[38] Bora E, Harrison BJ, Davey CG, Yucel M, Pantelis C (2012). Meta-analysis of volumetric abnormalities in cortico-striatal-pallidal-thalamic circuits in major depressive disorder. Psychol Med.

[39] Green MJ, Cahill CM, Malhi GS (2007). The cognitive and neurophysiological basis of emotion dysregulation in bipolar disorder. J Affect Disord.

[40] Strakowski SM, Adler CM, Holland SK, Mills NP, DelBello MP, Eliassen JC (2005). Abnormal fMRI brain activation in euthymic bipolar disorder patients during a counting stroop interference task. Am J Psychiatry.

[41] Belmaker RH, Bersudsky Y (2004). Bipolar disorder: mania and depression. Discov Med.

[42] Scott J, Pope M (2003). Cognitive styles in individuals with bipolar disorders. Psychol Med.

[43] Johnson S, Tran T (2007). Bipolar disorder: what can psychotherapists learn from the cognitive research?. J Clin Psychol.

[44] Zeng L, Wang H, Hu P, Yang B, Pu W, Shen H, Hu D (2018). Multi-site diagnostic classification of schizophrenia using discriminant deep learning with functional connectivity MRI. EBioMedicine.

[45] Tao H, Guo S, Ge T, Kendrick KM, Xue Z, Liu Z, Feng J (2013). Depression uncouples brain hate circuit. Mol Psychiatry.

